# The Current and Future Challenges of Hip Fracture Management in Malaysia

**DOI:** 10.5704/MOJ.2011.004

**Published:** 2020-11

**Authors:** T Ong, HM Khor, CS Kumar, S Singh, EGM Chong, K Ganthel, JK Lee

**Affiliations:** 1 Department of Medicine, University of Malaya, Kuala Lumpur, Malaysia; 2 Department of Orthopaedic Surgery, University of Malaya, Kuala Lumpur, Malaysia; 3 Department of Geriatric Medicine, Hospital Kuala Lumpur, Kuala Lumpur, Malaysia; 4 Department of Orthopaedics, Hospital Kuala Lumpur, Kuala Lumpur, Malaysia; 5 Department of Orthopaedics, Beacon Hospital, Petaling Jaya, Malaysia

**Keywords:** hip fracture, fragility fracture, osteoporosis, aged

## Abstract

By 2050, it is predicted that six million hip fractures will occur each year of which the majority will happen in Asia. Malaysia is not spared from this predicted rise and its rate of increase will be one of the highest in this region. Much of this is driven by our unprecedented growth in the number of older people. Characteristics of individuals with hip fractures in Malaysia mirror what has been reported in other countries. They will be older multimorbid people who were already at risk of falls and fractures. Outcomes were poor with at least a quarter do not survive beyond 12 months and in those that do survive have limitation in their mobility and activities of daily living. Reviewing how these fractures are managed and incorporating new models of care, such as orthogeriatric care, could address these poor outcomes. Experts have warned of the devastating impact of hip fracture in Malaysia and that prompt action is urgently required. Despite that, there remains no national agenda to highlight the need to improve musculoskeletal health in the country.

## Introduction

Hip fracture is the most common fragility fracture in older people^1^. They occur after a low-trauma injury, usually after a fall from a standing height or less. It commonly indicates underlying osteoporosis. Hip fractures are associated with increased morbidity, disability and mortality^2,3^. Individuals with hip fractures utilise significant healthcare resources as their hospital treatment consumes large amount of inpatient bed-days, the majority require surgical fixation and many on discharge need some form of support for daily living^2,4^. Epidemiology data has projected a staggering increase in the number of hip fractures over the coming decades. It is predicted that by 2050 there will be up to 6 million hip fractures occurring each year, and half of them will be in Asia due to the rapid population aging in this part of the world^5^.

Similar to many other countries in this region, the speed of increase in both proportion and absolute numbers of older people in Malaysia is unprecedented^6^. In 2010, only 5% were 65 years and over, but by 2040 this will increase to 14.5%^7^. This equates to an increase from 1.4 million older person in 2010 to 6 million in 2040. Many of our older people will also be living longer with the average life expectancy projected to increase from 74 years to 81 years by 20508. Hence, many clinical experts have already warned of the increase in number of hip fractures and its future impact on health services in Malaysia^5,9,10^. This narrative review of existing literature on hip fracture in Malaysia will describe its current state and what is required to address it in the future.

## Hip Fracture Epidemiology in Malaysia

There is geographical variation in hip fracture epidemiology and local data is important to understand its clinical burden^11,12^. The first Malaysian report on hip fracture epidemiology described increasing hospital admission to a tertiary hospital, Hospital Kuala Lumpur from 1981 (48 per 100,000 population) to 1989 (70 per 100,000 population)^13^. Subsequently, the most comprehensive study on Malaysia’s hip fracture epidemiology was reported from 56 public and private hospitals between 1996 to 1997. Its overall incidence was 90 per 100,000 population, among individuals aged 50 years and above^14^. Incidence did not change between those two years. It was higher among women (women, 140 per 100,000 population vs men, 65 per 100,000 population) and older age (≥75 years, 495 per 100,000 population vs 50-54 years, 10 per 100,000 population)^14^.

A report by the Asian Federation of Osteoporosis Societies have projected that by 2050 there will be a 3.5-fold increase in the number of hip fractures, from 6000 to almost 21,000 fractures occurring annually, costing over USD125 million (MYR540 million) each year in healthcare expenditure^5^. Malaysia’s expected rate of increase will be the highest among countries in this region^5^.

## Characteristics of Individuals with Hip Fracture

Characteristics of individuals that sustained a hip fracture in Malaysia do not differ much from what has been reported elsewhere. These individuals were older, multimorbid, mostly female and were already at risk of falls and fractures. In Malaysia, the average age ranged from 74-79 years^15-19^, up to 75% were female^14-18,20^, 77% had at least one comorbid illness^15,16,18^ and 26% were considered multimorbid^18^. Of the most common comorbid illnesses, the prevalence of diabetes mellitus was reported between 25-46%^15,16,21^, hypertension between 34-74%^15,16,21^, ischaemic heart disease between 311%^15,16^, and stroke between 6-10%^15,16,21^. A quarter of individuals with hip fractures already reported at least one previous fall^17^, 13% a previous fragility fracture^20^, and 5% a previous hip fracture^22^. Despite the high fracture risk, only 12% were reported to be taking medication to optimise their bone health (anti-osteoporosis medication, calcium or vitamin D supplementation)^22^. Differences based on ethnicity were also reported, where most hip fractures occurred among Chinese (40-78%), Malay (8-44%) and followed by Indian ethnicity (12-20%)^14-16,18-20^. Most individuals were still living at home (95%) at the time of their fracture^15^. However, this did not mean they were fully independent. At least 30% required assistance with mobility, and 5% were bedbound^15,17,21^. Thirty percent were dependent for activities of daily living^15,18^.

## Hip Fracture Presentation and Treatment

Falls were the most common cause for hip fractures, ranging from 83-100%^15,18,21,22^. Almost all presented on the day of the injury^17^. Regarding types of fracture, femoral neck fractures were seen in 23-62%, and inter-trochanteric fractures in 2646% of individuals^18-21^.

The American Society of Anaesthesiologists (ASA) classification was the most common risk assessment tool used pre-operatively^18,22^. Most individuals were surgically managed, and this was reported between 67-100%^17,19,21,23^. ASA 1 and 2 made up almost 85% of these patients^18,22^. Less than 20% were operated within the recommended treatment threshold of 48 hours from admission^17,18,23^. The median time to surgery was five days^17,18^. The most common reason for delay was the lack of operating theatre capacity (28-49%) and the need to medically optimise these patients (2328%)^17,22,23^. Other reasons reported were patients being on an antiplatelet or anticoagulant, and financial issue^17,22,23^. Among those that were not operated, at least a third was because the individual refused surgery^22,23^. Difference in characteristics and presentation between those operated and not operated have not been reported in Malaysia. No studies have also been performed to look at factors influencing decision to operate.

Falls and osteoporosis are two modifiable risk factors for subsequent fractures. However, both were not routinely addressed in hip fracture individuals. In one study, none had a falls assessment^23^. Only between 8-44% of individuals post-hip fracture were initiated on anti-osteoporosis medication^20,23,24^. When it was initiated, one study demonstrated low persistence over four years, with the median duration of individuals taking oral bisphosphonate was one month^20^. Fracture Liaison Services (FLS) is a secondary fracture prevention service which proactively identifies and treats individuals at risk of future fragility fractures. It is a highly effective model of care to optimise bone health^25^.

## Outcomes Among Older People with Hip Fractures

The most reported outcome was inpatient mortality which ranged between 0.4-5%^17,20,21,22^. A number of risk factors were associated with inpatient mortality, such as increasing age, hearing impairment, visual impairment, chronic kidney disease, thyroid disease and an abbreviated mental test of less than eight^17,21^. Those not operated were 2.6 times more likely to die compared to those operated^21^.

Mortality was also reported at 30-days (7-10%)^23,26^, 6 months (14-22%)^18,26^, and 12 months (26%)^18^. 12-month mortality was higher among those with longer time to surgery from admission and more dependent for activities of daily living pre-fracture^18^. There was a trend towards higher mortality in men and higher ASA grade (ASA 3 and 4)^18^. The type of fracture was not associated with mortality^18,21^.

Length of stay ranged from 7-17 days^18,20,22,23^. Only one study reported discharge destination. The majority returned to their own home (89%) but there was no mention of care needs^22^. Most individuals with hip fracture sustained a deterioration in mobility and became more dependent for daily living, which persisted till 6 to 12 months post-fracture. In one study, all individuals with hip fractures required a walking aid on discharge (70% walking frame, 28% wheelchair and 1% bedbound)^20^. At six months post-discharge, only 24% were independently mobile, 14% were immobile and the rest required a walking aid^26^. Most did not regain their pre-fracture mobility^26^. At 12 months post-hip fracture, one study reported that 59% were dependent for their personal activities of daily living^18^.

## National Hip Fracture Registry

A number of national hip fracture registries exist, such as in England and Wales^27^, Scotland^28^, and in Australia and New Zealand^29^. These registries collect data on patient demographics, fracture details, surgical treatment, multidisciplinary input, fracture risk reduction strategies, post-discharge outcomes and resources available to manage hip fractures in their respective countries. Regular reports are published. It allows individual hospitals to understand the care they are currently delivering and benchmark standards with other hospitals and quality standards. This drives improvement in care.

The National Orthopaedic Registry of Malaysia (NORM) is a clinical database which aims to report on orthopaedic services in public hospitals throughout Malaysia. It is affiliated with the Association of Clinical Registry of Malaysia. The first phase of the registry focussed on hip fractures and the NORM hip fracture report 2009 was published in 201022. It collated data in 2008 from 18 public hospitals contributing 794 patient-level data into its database^30^. The registry has been utilised as a tool to understand the hip fracture problem from a local perspective^15,2^1. Since the initial report, no further hip fracture publication has been made available in the public domain.

## National Musculoskeletal Health Agenda

Osteoporosis, fragility fractures and musculoskeletal health is not part of any national health priorities^8,10^. Even in the Ministry of Health’s 2016 strategic plan to address the rise of non-communicable diseases, bone health was not listed as a priority area^31^. This despite the musculoskeletal agenda being highlighted as important by the World Health Organisation in their report on ageing and health^32^. Falls and fractures are complex health issues prevalent in older age that have serious consequences and can be prevented.

The Malaysian Osteoporosis Society have published its clinical guidance to support management of osteoporosis to prevent the onset of fractures^33^. The Fragility Fracture Network of Malaysia is another multidisciplinary specialist society with the aim of improving fracture care and better secondary prevention^34^. Both are strong advocates for better musculoskeletal health in older people despite the lack of a national agenda for now.

## Moving Forward: A Discussion of Current and Future Challenges

The ageing Malaysian population will see an increase in the number of hip fractures in Malaysia. Many will have issues with multimorbidity. Hence, this group of older people do not do well as a consequence of their fracture. At least a quarter will not survive beyond 12 months, and even those that do will have limitation in their mobility and daily living. The acute healthcare costs and subsequent cost of providing care will be significant.

In light of this, we need to consider a change in how hip fractures should be managed in hospitals to improve its poor outcomes. Older people with hip fractures need more than just their hip fracture fixed. Their concomitant medical issues, pre-operative optimisation, post-operative rehabilitation, minimising hospital acquired complications and reduce their risk of future fractures needs addressing. For instance, at best only one in five patients were operated within 48 hours from admission, the threshold that has been shown operating after increases mortality and lead to worse outcomes^35^.

Therefore, many stakeholders in hip fracture care have embraced the medical-surgical model known as orthogeriatric care. It works on the principle of shared patient ownership between an orthopaedic team and a medical team led by a specialist physician in older people (geriatrician) to address the individual’s multiple health issues. Orthogeriatric care has been shown to reduce medical complications, hospital mortality by 40%, lower time to surgery and less functional deterioration^36-38^. This collaborative model of care has been adopted as the expected standard of care in international hip fracture guidelines^27-29^. Currently, three hospitals in Kuala Lumpur are known to deliver orthogeriatric care. However, it remains uncertain how this model of care should be ideally operationalised and its effectiveness within Malaysia’s health system. As the patient characteristics and issues are similar to what has been reported elsewhere, there is no reason similar benefits would not be seen.

Another key consideration moving forward is the implementation of FLS services on a much wider scale in Malaysia. A fragility fracture increases the risk of another fracture. Hence, preventing further falls and fractures needs to be embedded into routine care for hip fracture individuals. FLS service's effectiveness is supported by a robust evidence based which has demonstrated reduction in subsequent fractures and mortality by up to 50% and 35% respectively^39^. Currently, there are three FLS services in Malaysia and they are located in urban Klang Valley^25^.

There remain huge gaps in the current literature around hip fracture care in Malaysia. Information on peri-operative care, surgery and rehabilitation intervention is lacking. Where there is scientific literature, most studies were based in Kuala Lumpur where its urban population demographics is different to other areas of Malaysia^40^. Almost all studies were performed in public hospitals, with little from the private sector. Hence, potentially providing only a singular view of hip fracture in Malaysia. It is likely differences in patient characteristics and hip fracture treatment exist in these two sectors similar to what has been seen in the treatment of other healthcare conditions, e.g. cancer and antibiotic prescribing^41,42^.

Moving forward, a change in the way we address the hip fracture problem in Malaysia is required. A review of our hip fracture treatment is needed, a research agenda prioritised. and acknowledgement at a national level urgently insisted. Whenever the fracture issue has been acknowledged, especially as part of a national agenda, better outcomes were seen^43,44^.
